# Establishment and Validation of a Tumor Microenvironment Prognostic Model for Predicting Bladder Cancer Survival Status Based on Integrated Bioinformatics Analyses

**DOI:** 10.1155/2022/4351005

**Published:** 2022-10-03

**Authors:** Qiu Chen, Guicao Yin, Xingjun He, Tianlin Jiang, Haisen Zhou, Yunjiang Wu, Yifan Li

**Affiliations:** ^1^Department of Urology, The Affiliated Hospital of Yangzhou University, Yangzhou University, Yangzhou 225007, Jiangsu, China; ^2^Medical College, Yangzhou University, Yangzhou 225009, Jiangsu, China; ^3^Nanjing Lishui District Hospital of Traditional Chinese Medicine, Lishui Hospital of Chinese Medicine, Affiliated to Yangzhou University Medical College, Nanjing 211299, Jiangsu, China; ^4^Department of Thoracic Surgery, The Affiliated Hospital of Yangzhou University, Yangzhou University, Yangzhou 225007, Jiangsu, China

## Abstract

This study was designed to analyze the characteristics of bladder cancer-related genes and establish a prognostic model of bladder cancer. The model passed an independent external validation set test. Differentially expressed genes (DEGs) related to bladder cancer were obtained from the Gene Expression Omnibus (GEO), The Cancer Genome Atlas (TCGA), and Genotype-Tissue Expression (GTEx) databases. WGCNA was used to fit the GSE188715, TCGA, and GTEx RNA-Seq data. Fusing the module genes with the high significance in tumor development extracted from WGCNA and DEGs screened from multiple databases. 709 common prognostic-related genes were obtained. The 709 genes were enriched in the Gene Ontology database. Univariate Cox and LASSO regression analyses were used to screen out 21 prognostic-related genes and further multivariate Cox regression established a bladder cancer prognostic model consisting of 8 genes. After the eight-gene prognostic model was established, the Human Protein Atlas (HPA) database, GEPIA 2, and quantitative real-time PCR (qRT-PCR) verified the differential expression of these genes. Gene Set Enrichment Analysis and immune infiltration analysis found biologically enrichment pathways and cellular immune infiltration related to this bladder cancer prognostic model. Then, we selected bladder cancer patients in the TCGA database to evaluate the predictive ability of the model on the training set and validation set. The overall survival status of the two TCGA patient groups in the training and the test sets was obtained by Kaplan–Meier survival analysis. Three-year survival rates in the training and test sets were 37.163% and 25.009% for the low-risk groups and 70.000% and 62.235% for the high-risk groups, respectively. Receiver operating characteristic curve (ROC) analysis showed that the areas under the curve (AUCs) for the training and test sets were above 0.7. In an external independent validation database GSE13507, Kaplan–Meier survival analysis showed that the three-year survival rates of the high-risk and the low-risk groups in this database were 56.719% and 76.734%, respectively. The AUCs of the ROC drawn in the external validation set were both above 0.65. Here, we constructed a prognostic model of bladder cancer based on data from the GEO, TCGA, and GTEx databases. This model has potential prognostic and clinical auxiliary diagnostic value.

## 1. Introduction

Bladder cancer (BLCA) is a common malignant tumor, and its morbidity and mortality rank first among urinary system tumors [1, 2]. BLCA is divided into bladder urothelial carcinoma and bladder nonurothelial carcinoma, with the former accounting for most instances of BLCA. Urothelial carcinomas are divided into bladder, renal pelvis, ureter, and proximal urethra cancers. BLCA accounts for 90 to 95% of urothelial cancers. Histologically, nonmuscle-invasive BLCA accounts for 75% of BLCA, and muscle-invasive or metastatic accounts for 25% [3]. The complexity of cancer types increases the difficulty of BLCA diagnosis and treatment [4]. Although the overall 5-year survival rate of nonmuscle-invasive BLCA can reach 90%, most patients require lifelong cystoscopy and combined intervention therapy, making BLCA one of the most expensive cancers to treat [5]. The 5-year survival rates for muscle-invasive and metastatic BLCA are 36% and 5%, respectively [6]. In Europe, 5-year standard relative survival rates for BLCA vary widely [7]. Most developing countries lack survival statistics based on large-scale populations, so it is necessary to identify new biomarkers and establish a relatively complete prognostic model for BLCA diagnosis and treatment.

Risk stratification is an effective tool for cancer management, and reasonable risk stratification can increase the use of correct interventions for high-risk groups and reduce unnecessary interventions for low-risk groups [8, 9]. In recent years, risk grading guidelines have been continuously adjusted to improve the accuracy of daily clinical use and reduce the complexity of practical operations [10–13]. Compared with traditional tumor clinical parameters, risk stratification based on molecules and factors can better evaluate the scores of in situ immune cell infiltration and abnormal DNA and mRNA levels in tumors [14–16].

High-throughput technology is an effective means to measure the degree of molecular influence on tumors [17, 18]. RNA-Seq transcriptome sequencing, the primary high-throughput technology tool for exploring transcriptomic information, has been widely used in cancer research. Indeed, a large proportion of cancer research relies on advanced RNA-Seq sequencing technologies and their continual improvement [19]. At present, a comprehensive prognostic model of BLCA based on RNA-Seq data is lacking. This study was designed to determine the prognostic significance of transcriptomic information in BLCA through screening integrated high-throughput RNA-Seq data in the Gene Expression Omnibus (GEO) [20], The Cancer Genome Atlas (TCGA) [21], and Genotype-Tissue Expression (GTEx) databases [22, 23]. Using this approach, prognostic-related genes in normal and tumor tissue were identified. These genes were further narrowed down to identify genes significantly associated with overall survival to establish a prognostic risk stratification scoring model. The model was evaluated on an external validation dataset. The prognostic model for patients with BLCA, established in this study, has potential prognostic value.

## 2. Materials and Methods

### 2.1. Experimental Design and Cohort Study

BLCA and normal bladder tissue transcriptome and clinical data were obtained from GEO, TCGA, and GTEx databases. A systematic retrospective analysis was used to avoid bias caused by a single cohort or small sample cohort. Two groups of DEGs were identified by comparing RNA-Seq transcriptome data of BLCA tissue and normal bladder tissue. Then, weighted gene coexpression network (WGCNA) analyses [24] of BLCA tissue and normal bladder tissue were performed in the GEO, TCGA, and GTEx databases to identify the relationship between BLCA gene expression profiles and clinical BLCA manifestations. WGCNA results focused on the role of significant genes in predicting the prognosis and survival status of patients with BLCA. The common genes identified in the DEGs and WGCNA analyses were considered prognostic-related genes and were subjected to univariate Cox regression analysis. The BLCA samples in TCGA were randomly divided into training and validation sets (*n* = 203 each), and the training set was used to optimize the LASSO coefficient, perform multivariate Cox regression analysis, and establish a risk scoring model. LASSO regression analysis [25] was used to narrow down the prognostic-related gene results to avoid overfitting and to replace highly correlated genes. Finally, multivariate Cox regression analysis was used to process the screened genes, and a prognostic model was established to predict the overall survival rate.

After the prognostic model was established, GEPIA 2, HPA, and qRT-PCR were used to validate the significance of genes in the prognostic model. GSEA and immune infiltration analysis divide all tumor samples in TCGA into high- and low-risk groups based on risk scores, showing results of enrichment pathways and cellular immune infiltration.

The validation set was used to verify the model's accuracy for risk stratification and prognostic survival. GSE13507 was used for independent external validation of model validity as external validation. The flow chart of this study is shown in Supplementary Figure 1.

### 2.2. Data Acquisition

The RNA-Seq transcriptome sequencing data of BLCA tissue and normal tissue were downloaded from the GEO (GSE188715 and GSE13507). The RNA-Seq transcriptome sequencing data and clinical information of BLCA patients and normal samples were downloaded from the GTEx [22, 23] and TCGA [21, 26] databases using the Xena platform [27].

### 2.3. Identification of Differentially Expressed Genes from GEO Sequencing Data, TCGA, and GTEx Sequencing Data

After downloading the GEO data, we found that the GSE188715 dataset had been preprocessed by the uploader, and DEGs were filtered using |Log2FC| > 1 and *P* <  0.05 as cut-off values. GSE13507 was used as an external validation set for the prognostic model due to having sufficient cancer and clinical data. Gene expression data used above was assessed by Illumina Human-6 v2.0 Expression BeadChip and DNBSEQ-G400 platforms. After RNA-Seq data was downloaded, FPKM values from GTEx were processed by Log2(*x* + 0.001), and FPKM values from TCGA were processed by Log2(*x* + 1). The format of both TCGA and GTEx datasets was unified as Log2(*x* + 1), and the *R* package “limma” [28] was used for normal bladder tissue and BLCA tissue with a cut-off value of |Log2FC| > 1 and *P* <  0.05. Log2FC > 0 indicates that the gene is overexpressed in tumor tissue.

### 2.4. Weighted Gene Coexpression Network Analysis

The *R* package “WGCNA” was called in GSE188715, TCGA, and GTEx, respectively, as the detection gene module, and the correlation of each module with the occurrence of cancer was evaluated. The specific steps used for the two WGCNA analyses were as follows: (a) extract the gene expression profiles of BLCA patients and normal bladder tissue from GSE188715, TCGA, and GTEx, respectively; (b) perform sample clustering to detect any outliers; (c) select the best scale-free topological fit index (soft threshold) to achieve a higher average network connectivity with a scale-free fit number greater than 0.9; (d) transform the adjacency matrix into a topological overlap matrix (TOM) to define gene coexpression similarity; (e) based on the dissimilarity measured by TOM, use the “hclust” algorithm to perform gene hierarchical clustering; (f) set the optimal module size, and identify the modules using dynamic tree-cutting; (g) calculate dissimilarity of the module eigengenes and observe the degree of similarity to build module; (h) on the basis that the characteristic gene expression profile of each module had been determined, determine the characteristic genes of the modules with significance.

### 2.5. GO Analysis

After WGCNA module clustering of the two groups of data, the significant module genes with the largest and smallest module significance in the two WGCNA module groups were selected, and these significant genes were intersected with the GEO, TCGA, and GTEx DEGs to identify the common prognostic-related genes. GO analysis was performed on the common prognostic-related genes in BLCA tissue and normal bladder tissue of GSE188715, TCGA, and GTEx, using the *R* package “clusterProfiler” [29] with adjusted *P* <  0.01 and *Q* < 0.01 as cut-off values. The *R* package “GOplot” was used to produce GO enrichment maps. Three types of enrichment maps were drawn: biological process (BP), cell component (CC), and molecular function (MF).

### 2.6. Establishment of a Prognostic Model

The common prognostic-related genes were entered into the *R* package “survival” to construct a prognostic model. The screening criteria were as follows: hazard ratio and 95% confidence interval >1 (genes affecting overall survival). The screened genes were subjected to univariate Cox regression analysis, with *P* <  0.01 as the significance threshold [30]. These significant genes were further narrowed down by LASSO regression analysis using the *R* package “glmnet”, and the optimal tuning parameter (*λ*) was selected to achieve the smallest partial likelihood deviation in the cross-validation plot. Genes with corresponding coefficients not equal to 0 were entered into the multivariate Cox regression model. The *R* package “caret” [31] was used to randomly divide TCGA BLCA samples into training and validation sets (*n* = 203 each). Finally, train the model in the training set. The expression level of the final screened gene was multiplied by its Cox regression coefficient, and these values were added to calculate the risk score [30].

### 2.7. Validation Genes in Prognostic Model by GEPIA 2 and HPA Database

GEPIA 2 was used to verify the expression difference of prognostic model genes in bladder cancer tumor samples and normal samples [32]. The RNA-Seq datasets used in the GEPIA 2 are based on UCSC Xena (http://xena.ucsc.edu), which is computed by standard pipelines to analyze RNA-sequencing expression of tumor and normal samples from the TCGA and GTEx datasets. Therefore, this study used the TCGA and GTEx gastric cancer RNA-Seq data integrated by the GEPIA 2 platform for comprehensive validation. With |Log_2_FC| cutoff = 1, *p* value cutoff = 0.01, draw a box plot of the RNA-Seq data of bladder cancer genes in the prognostic model.

The Human Protein Atlas (HPA) database [33] uses transcriptomic and proteomic technologies to study protein expression in tumors and normal tissues of various human organs. The immunohistochemistry (IHC) staining data for this study were downloaded from the HPA database. Then, the results of bladder cancer pathology and normal bladder tissue were processed.

### 2.8. Validation of Two Most Significant Genes in Clinical Patients' Tissue

Among all genes, *GNG7* and *MXRA7* have the two highest coefficients in the prognostic model, indicating that they influence the prognosis of BLCA greatly. 24 pairs of BLCA tumor tissues and adjacent normal mucosa tissues were collected from the Affiliated Hospital of Yangzhou University and the Peking University First Hospital (Supplementary Table. 1). The protocol was approved by the Institutional Ethical Review Board of Yangzhou University. Total RNA was extracted from samples with RNA Extraction TRIzol (Life, Shanghai, China). Reverse transcription and quantitative real-time PCR (qRT-PCR) was conducted by TransScript Green One-Step qRT-PCR SuperMix (Transgen, Beijing, China). The reaction conditions of the Agilent AriaMx quantitative real-time PCR instrument are as follows: Step 1: 1 cycle of 95°C for 30 seconds; Step 2: 40 cycles of 95°C for 5 seconds, 60°C for 30 seconds; Step 3: dissolution curve. Primers are available in Table 1. Relative quantitation analysis of two-gene expression data was conducted according to the 2^−ΔΔCT^ method.

### 2.9. Gene Set Enrichment Analysis (GSEA)

The risk score of each patient in TCGA is calculated based on the prognostic model. Then, all patients were divided into high- and low-risk groups based on the median risk score. We extracted TCGA data as an expression matrix. KEGG gene sets in MSigDB [34] were set as functional annotation gene sets. The GSEA analysis [35] of bladder cancer proceeded.

### 2.10. Immune Infiltration Analysis in Immune Cells

We uploaded the genetic data of TCGA bladder cancer patients on the TIMER platform [36] and download the immune infiltration data of B cell, T CD4+ cell, T CD8+ cell, myeloid dendritic cell, macrophage, and neutrophil on the platform. The Pearson correlation analysis uncovered the relationship between high- and low-risk groups and immune scores based on these immune infiltration data.

### 2.11. Prognostic Model Training and Validation Sets

To train and validate the prognostic, we calculate the risk score of each patient in training and test sets. Patients were, respectively, divided into high- and low-risk groups based on the median training and test set risk scores. The *R* package “survival” was used to calculate the final prognosis of one- and three-year overall survival by Kaplan-Meier survival analysis. The *R* package “survivalROC” [37] was used to plot the ROC curve of the training and test set, determining patient one- and three-year survival accuracy. GSE13507 was used to externally validate the prognostic model validity.

## 3. Results

### 3.1. GEO, TCGA, and GTEx RNA-Seq Transcriptome Database Analysis

GSE188715, downloaded from the GEO database, contains data from 57 cases of cancer and 13 normal bladder tissue samples. The TCGA database contains information from 406 cancer samples and 19 normal bladder samples. Due to the lack of normal bladder tissue sample data in TCGA, data from 9 normal bladder tissue samples were downloaded from the GTEx database maintained by MIT and Harvard University, increasing the number of normal bladder tissue samples in TCGA and GTEx to 28 cases. GTEx and TCGA databases RNA-Seq sequencing data and clinical characterization information were downloaded from the UCSC Xena platform (http://xena.ucsc.edu/).

The *R* package “limma” was used to analyze cancer data and normal bladder tissue data from GEO, TCGA, and GTEx databases. Using the cut-off value of |log2FC| > 1 and *P* < 0.05, a total of 4183 DEGs were screened in GSE188715. Of these 4183 DEGs, 2082 genes were upregulated and 2101 genes were downregulated in cancer tissue relative to normal tissue. There were 1990 DEGs in TCGA and GTEx. Of these 1990 DEGs, 843 were upregulated and 1147 were downregulated genes in cancer tissue compared with normal tissue.

### 3.2. Identification of High-Associated Genes in Bladder Carcinogenesis in WGCNA

To find the correlation between cancer occurrence and genes, we used the *R* package “WGCNA” to fit the highly correlated genes in GSE188715, TCGA, and GTEx into modules and analyzed their connection with the occurrence and development of BLCA. Due to there being sufficient cases and gene data in the GEO, TCGA, and GTEx databases to meet WGCNA analysis conditions, gene maps of BLCA patients and noncancer populations meeting the requirements were extracted from the database. A sample dendrogram was plotted for GSE188715, and a threshold of 4 was determined (Supplementary Figure 2(a)). Dynamic tree-cutting with a module size of 30 resulted in 33 color-coded modules based on topological overlap matrix gene clustering (Supplementary Figure 2(b)). A TCGA and GTEx sample dendrogram was plotted, and a threshold of 3 was determined (Supplementary Figure 3(a)). Dynamic tree-cutting with a module size of 30 resulted in 42 color-coded modules based on topological overlap matrix gene clustering (Supplementary Figure 3(b)).

**Figure 1:**
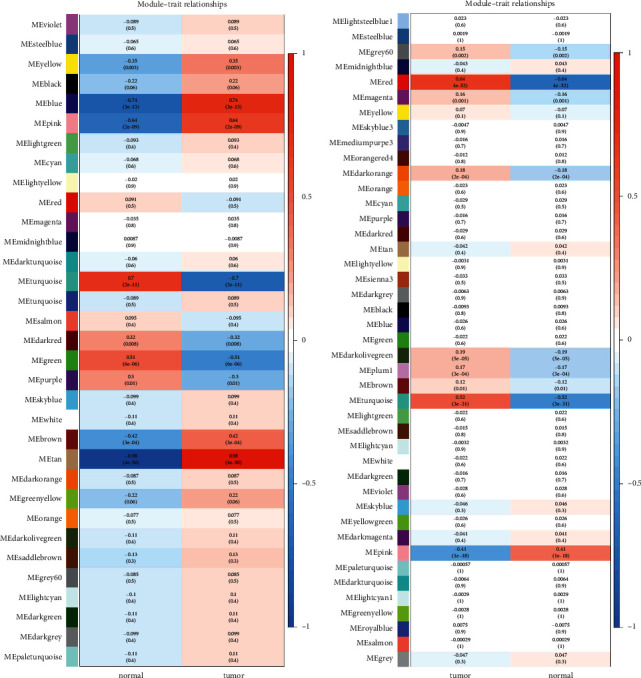
Module-trait relationships in GSE188715, TCGA, and GTEx. (a) WGCNA labeled heatmap for GSE188715. (b) WGCNA labeled heatmap for TCGA and GTEx. In the labeled heatmaps, each row represents a module characteristic gene encoded by color, and the two columns represent clinical characteristics of tumor and normal tissue, respectively. Each cell represents the Pearson correlation coefficient and P value (in parentheses) of the corresponding module characteristics, and the color of each cell represents the value of correlation.

The relationship between the gene module and the development of cancer should be sought. WGCNA analysis in GSE188715 revealed that the tan module had the highest positive tumor correlation (*r* = 0.98, *P*=4*e* − 50), and the turquoise module has the lowest negative tumor correlation (*r* = −0.7, *P*=2*e* − 11) (Figure 1(a)). WGCNA analysis in TCGA and GTEx revealed that the pink module has the highest positive tumor correlation (*r* = 0.41, *P*=1*e* − 18), and the red module has the lowest negative tumor correlation (*r* = −0.64, *P*=4*e* − 52) (Figure 1(b)). Therefore, this study used the genes in these four modules and two sets of DEGs to construct a prognostic model. The study firstly screened 709 prognostic-related genes among two groups of WGCNA gene clustering and two DEGs results.

### 3.3. GO Analysis Results

**Figure 2:**
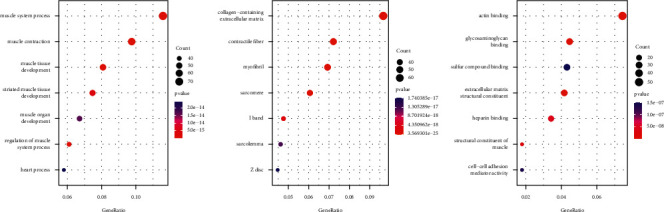
The seven most significantly enriched pathways in BP, CC, and MF. The size of the circle indicates the number of enriched genes, and the color corresponds to the adjusted *P* value.

GO analysis showed that the 709 prognostic-related genes had 334 pathways in biological process (BP) (Figure 2(a)), 52 pathways in cellular component (CC) (Figure 2(b)), and 12 pathways in molecular function (MF) (Figure 2(c)). The main involvement of the muscle system in biological processes is related to the function of the extracellular matrix tissue. Cell composition and molecular functions are mainly related to the collagen-containing extracellular matrix and actin binding.

### 3.4. Establishment of the BLCA Prognostic Model

Univariate Cox regression analysis was performed on the clinical manifestation association data to identify the predictive power of these 709 genes. The analysis conditions were hazard ratio and 95% confidence interval >1 and *P* < 0.01. Finally, 95 genes were obtained in the cohort, and correlations were predicted (Supplementary Figure 4).

**Figure 3:**
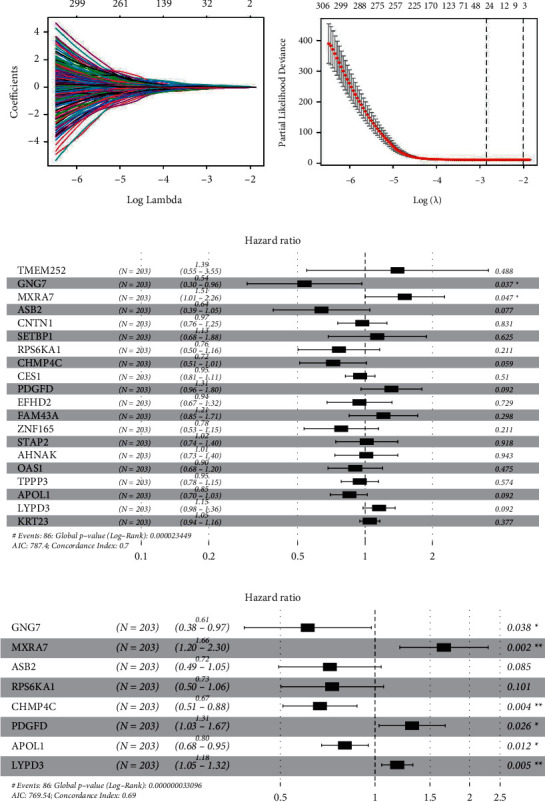
LASSO regression model and multivariate regression analysis results. (a) LASSO distribution of 95 differentially expressed genes associated with prognosis of bladder cancer. Each curve represents a coefficient *λ*. When it changes, the coefficient not 0 changes and enters the lasso regression model. (b) Selecting the best tuning parameters (*λ*) and cross-validation; red dotted line represents the best logarithm *λ*. When crossing, it is equivalent to the minimum value of the multivariate Cox model, and the two dotted lines represent one standard deviation of the minimum distance. (c) Hazard ratios and confidence intervals of 21 genes in LASSO regression analysis. (d) Multivariate Cox regression analysis results generated using the training set.

LASSO regression analysis was used to select genes with key predictive functions. In LASSO-penalized Cox regression, as log *λ* (tuning parameter) is changed, the corresponding coefficients of identified genes are reduced to 0. The tapered parameters suggest that their effect on the model can be ignored (Figure 3(a)). Then, in cross-validation, 21 genes reached the minimum value of partial likelihood bias, so at this point, log *λ* was close to −2.765 and 21 genes showed a certain effect. All genes showed a positive risk ratio (hazard ratio >1), revealing a positive effect on the development of BLCA (Figure 3(b)). Therefore, these 21 genes were fitted to the prognostic model. These genes are *THEM252*, *PCOLCE2*, *GNG7*, *MXRA7*, *ASB2*, *CNTN1*, *SETBP1*, *RPS6KA1*, *CHMP4C*, *CES1*, *PDGFD*, *EFHD2*, *FAM43A*, *ZNF165*, *STAP2*, *AHNAK*, *OAS1*, *TPPP3*, *APOL1*, *LYPD3*, and *KRT23* (Figure 3(c)).

The 406 BLCA samples in the TCGA databases were randomly divided into training and validation sets (*n* = 203 each). The training set was used to establish a prognostic model, and the validation set was used to test the accuracy of the prognostic model. Multivariate Cox regression analysis of the training set identified eight genes as independent predictors, namely, *GNG7*, *MXRA7*, *ASB2*, *RPS6KA1*, *CHMP4C*, *PDGFD*, *APOL1*, and *LYPD3*.

(1) $$\begin{array}{c} \text{The final predicted risk score}=\;GNG7\;\times \;\left(-0.491\right)+\;MXRA7\;\times \;0.5063\;+\;ASB2\times \left(-0.330\right)+\;RPS6KA1\;\times \left(-0.313\right)+\;CHMP4C\times \left(-0.403\right)+\;PDGFD\times \;0.273\;+APOL1\;\times \left(-0.219\right)+\;LYPD3\times 0.164. \end{array}$$

These figures are the regression coefficients of the multivariate Cox regression analysis (Figure 3(d)).

### 3.5. Eight Genes Expression in GEPIA 2, HPA, and qRT-PCR

**Figure 4:**
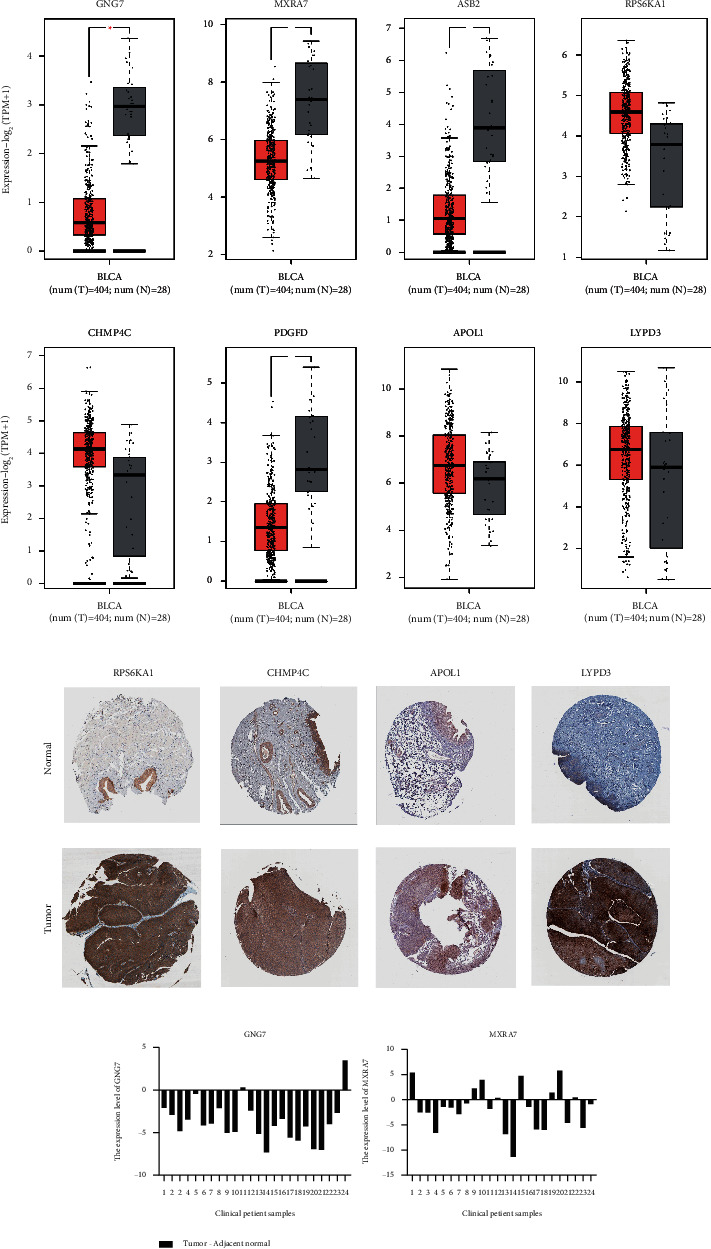
Validations in GEPIA 2 platform, HPA database, and qRT-PCR experiment. (a) The red and gray boxes represent cancer and normal tissues in TCGA and GTEx datasets, respectively. BLCA, bladder cancer, *p* < 0.01 (GEPIA 2 website). (b) Immunohistochemical staining of RPS6KA1, CHMP4C, APOL1, and LYPD3 in the Human Protein Atlas (HPA) database. (c) The expression level of GNG7 and MXRA7 in qRT-PCR (tumor expression minus normal expression).

The expression of eight genes in cancer and normal samples was validated in GEPIA 2. The box plot of GEPIA 2 presents the expression levels of the eight genes in the standard of expression-Log2 (TPM+1) (Figure 4(a)). We can find that the expression of GNG7, MXRA7, ASB2, and PDGFD in tumor samples is significantly lower than that in normal samples, while the expression of RPS6KA1, CHMP4C, APOL1, and LYPD3 is reverse.

This study also performed an IHC analysis in the bladder data of genes included in the prognostic model from the HPA database. The results of IHC staining are shown in Figure 4(b). The results showed that RPS6KA1, PDGFD, APOL1, and LYPD3 are all upregulated in tumor tissues, which were consistent with GEPIA2. Unfortunately, due to the novelty of prognostic genes in this model, data of another four genes could not be found in the HPA database.

After extracting RNA from tissues, the results of qRT-PCR were consistent with the results stated in the progress of filtering DEGs and GEPIA 2 analyses. In the 24 pairs of samples including tumor and normal tissues, most expressions of GNG7 and MXRA7 in tumor tissues minus their expressions in normal samples were less than 0, indicating that the two most significant genes have higher expressions in normal tissues (Figure 4(c)).

### 3.6. GSEA

**Figure 5:**
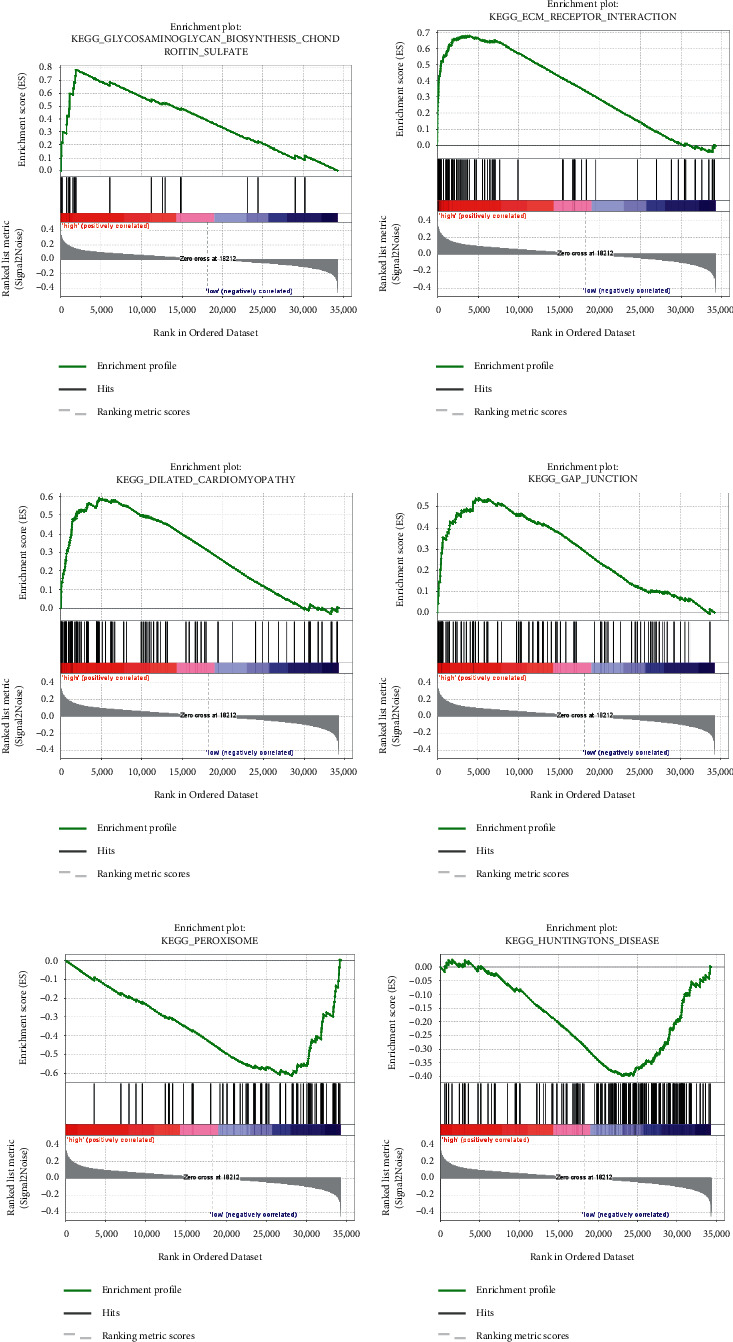
GSEA enrichment pathway analysis. (a–d) Top four significant enrichment pathways in high-risk groups. (e) (f) All significant enrichment pathways in low-risk groups.

The results of the GSEA analysis showed that there were 15 and 2 significantly enriched pathways (*P* <  0.05 and FDR <25%) in the high- and low-risk groups, respectively. In the high-risk group, enriched pathways involved glycosaminoglycan-biosynthesis-chondroitin-sulfate, ecm-receptor-interaction, dilated-cardiomyopathy, gap-junction, arrhythmogenic-right-ventricular-cardiomyopathy-arvc, focal-adhesion, vascular-smooth- muscle-contraction, melanoma, hypertrophic-cardiomyopathy-hcm, regulation-of-actin-cytoskeleton, renin-angiotensin-system, melanogenesis, neuroactive-ligand-receptor-interaction, calcium-signaling-pathway, and long-term-depression. The top four enriched pathways are presented in Figure5(a). In the low-risk group, enriched pathways related to peroxisome and Huntington's disease (Figure 5(b)). The prognostic model's risk score played a good classification role in the GSEA analysis based on the KEGG gene sets. The main significantly enriched pathways were clustered in the high-risk group. Targeted treatment of these enriched pathways may contribute to prolonging the prognosis and survival of bladder cancer patients.

### 3.7. Results of Immune Infiltration Analysis

**Figure 6:**
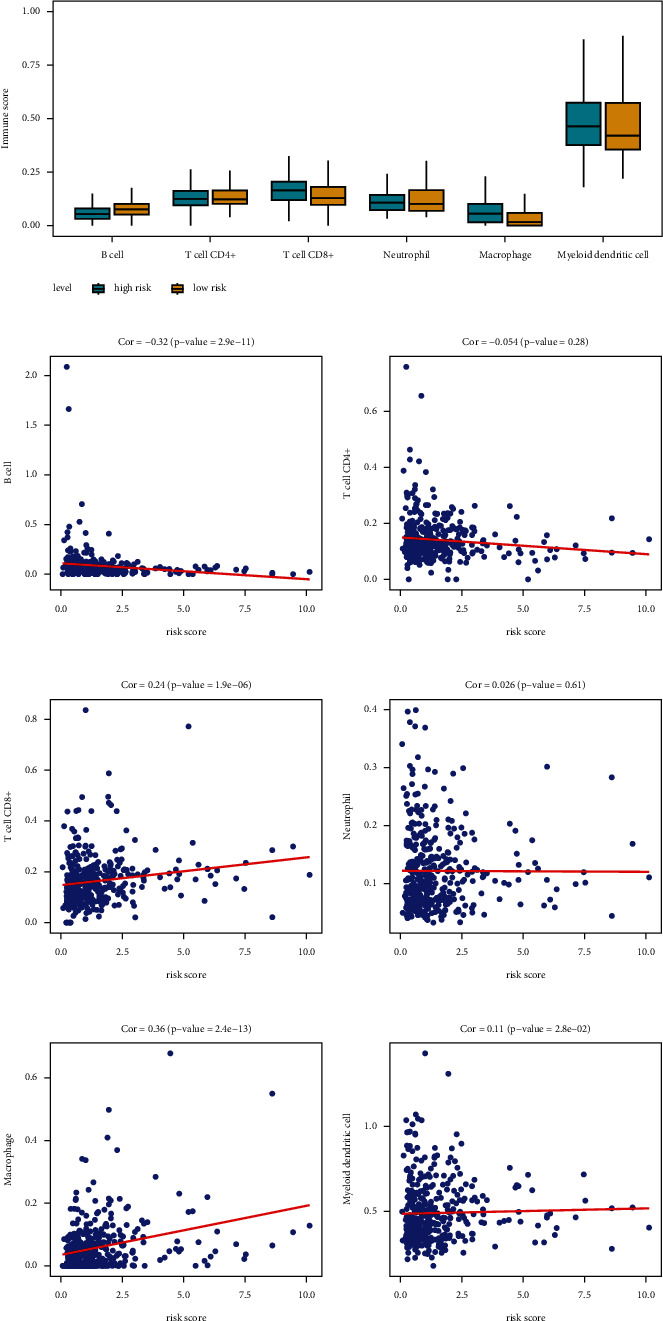
Cell immune infiltration analysis. (a) Immune scores of six immune cells (B cell, T CD4+ cell, T CD8+ cell, myeloid dendritic cell, macrophage, and neutrophil) in the high- and low-risk groups. (b–g) Correlation between risk score and six immune cells.

To explore the potential mechanisms of eight prognostic-related genes in the tumor microenvironment, this study performed a Pearson correlation analysis on six immune cells. The box plot showed that the high-risk group had higher immunes cores than the low-risk group in T CD8+ cell, macrophage, and myeloid dendritic cell (Figure 6(a)). In the B cell, the low-risk group had higher scores (Figure 6(a)). Correlation scatter plots showed that T CD8+ cell and macrophage had a significant positive relation (*P* <  0.05) with the prognostic model's risk scores (Figure 6(d), F), and the B cell had a significant negative relation (*P* <  0.05) with risk scores (Figure 6(b)).

### 3.8. Risk Stratification and Validation of the Prognostic Model Based on TCGA

**Figure 7:**
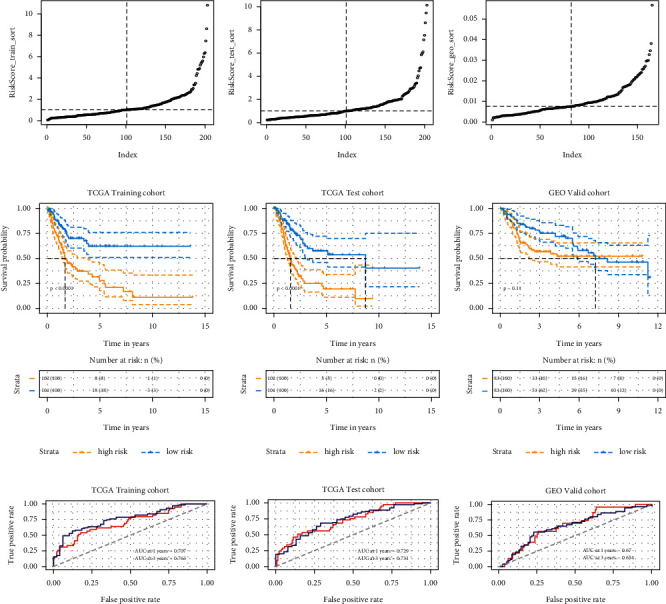
Expression of survival curves in prognostic models of TCGA bladder cancer patients and validation of model accuracy. (a–c) The risk score of patients in the training set, test set, and external validation set. The left side of the vertical dotted line represents low-risk patients, the right side represents high-risk patients, and the horizontal dotted line represents the critical value of the risk score used to define low- and high-risk patients. (d–f) Kaplan–Meier survival curve of patients in training, test set, and external validation set. (g–i) ROC curve of the training set, test set, and external validation set risk scores predicting one-year and three-year survival.

To verify the accuracy of the prognostic model, the study stratified the cancer data from the cancer TCGA database was stratified. Risk scores were first calculated for the TCGA training set; then, the training set was divided into high-risk (*n* = 102) and low-risk (*n* = 101) groups based on the median score, 1.03 (Figure 7(a)). The prognostic model identified the three-year survival rates of the high- and low-risk groups as 37.163% and 70.000%, respectively (Figure 7(d)). The high-risk group had a greater likelihood of low survival outcomes than the low-risk group. To determine the predictive accuracy of the prognostic model, a ROC curve analysis was performed. The one- and three-year survival AUCs in the training set were 0.70722 and 0.76282, respectively (Figure 7(g)).

The same method was used to test the accuracy of the prognostic model in the validation set, which was split into high- and low-risk groups (*n* = 102 each) using the median score of 1.002 (Figure 7(b)). This analysis also revealed that the high-risk group had a lower survival rate than the low-risk group. The three-year survival rates of the high- and low-risk groups were 25.009% and 62.235%, respectively (Figure 7(e)). ROC curve analysis revealed that the one- and three-year survival AUCs in the validation set were 0.72911 and 0.73118, respectively (Figure 7(h)). Therefore, this prognostic model successfully demonstrates potential predictive power for the populations at high- and low-risk groups of BLCA in the TCGA database.

### 3.9. External Validation of the Prognostic Model Based on GSE13507

GSE13507 was used as an external dataset to validate the accuracy of the prognostic model. GSE13507 data were stratified into high- and low-risk groups (*n* = 81 each) based on the median score of 0.007 (Figure 7(c)). This analysis showed that the GSE13507 high-risk group had a lower survival rate than the low-risk group. The three-year survival rates of the high- and low-risk GSE13507 groups were 56.719% and 76.734%, respectively (Figure 7(f)). ROC curve analysis revealed that the one- and three-year survival AUCs in the external validation set were 0.66993 and 0.65388, respectively (Figure 7(i)). This prognostic model demonstrated good predictive potential as shown using a GEO dataset for external validation.

## 4. Discussion

BLCA can occur at any age, is the most common malignant tumor of the urinary system, and is one of the ten most common tumors in the body. It is important to identify potential BLCA biomarkers and establish a prognostic model to improve prognosis. In this study, a BLCA prognostic model was developed based on gene sequencing data from the GEO database and transcriptome data from the TCGA database. The prognostic model contains eight genes (*GNG7*, *MXRA7*, *ASB2*, *RPS6KA1*, *CHMP4C*, *PDGFD*, *APOL1*, and *LYPD3*) to accurately calculate the risk score of BLCA patients. The model also consistently predicted lower overall survival in patients with high-risk scores.

In this study, eight bioinformatics tools were used to assess data downloaded from multiple databases: GO, GEPIA 2, GSEA, immune infiltration, WGCNA, univariate Cox regression, LASSO regression, and multivariate Cox regression analysis. GO analysis is a common gene enrichment analysis method used to classify genes according to their functions. It can reveal the functional characteristics of genes differentially expressed in BLCA and normal bladder tissue. GEPIA 2 is a tool for gene expression and survival analysis that integrates TCGA and GTEx databases. This study used this tool to draw box plots of 8 prognostic-related genes, which verified the expression differences of 8 genes in tumor tissue and normal tissue. As an enrichment method, GSEA can effectively make up for the omissions of some methods of “screening according to the fold difference threshold” and better reflect the significant functional differences caused by the accumulation of small changes in certain genes in a gene set. In some kind of situations where the differential expressions in certain genes are not so significant but do have obvious biological functions, this method of mining enrichment pathways helps us find comprehensive biological enrichment pathways. Immune infiltration analysis of six types of immune cells discovers the relationship between tumor prognosis and potential immune mechanism.

LASSO regression analysis is used to identify the core variables most relevant to prognosis and survival. This analysis is also used to optimize the model without reducing the clinical predictive ability and to reduce interference from variables unrelated to prognosis. WGCNA is a powerful bioinformatics tool used to detect gene clusters associated with clinical functions, identify clinically relevant gene markers, and group genes with similar clinical functions in the same module. The WGCNA analysis used in this study differs from the common clustering method by evaluating the correlation coefficient of BLCA gene expression values to the power of N, which is more consistent with scale-free network analysis and more in line with biological rules. Compared with common coexpression network analysis, this method includes the concepts of soft threshold and weight network to form a weighted coexpression network. The predictive power of multivariate Cox regression analysis is better than that of univariate Cox regression analysis because univariate Cox analysis is easily affected by the bias of univariate significant variables. The predictive power of multivariate regression to establish a prognostic model is better than that of univariate Cox regression [38]. Combining the use of eight bioinformatics tools to establish the BLCA prognostic model increases model accuracy, reduces model-independent variables, and improves predictive sensitivity and depth.

In this study, the modules closely related to the characteristics of BLCA were screened using WGCNA, and eight genes central to BLCA development were obtained (*P* <  0.05). Established prognostic models of BLCA have been described. Yao Kang et al. established a 13-gene prognostic model based on the prognostic analysis of BLCA samples [39], Zihao Chen et al. established a two-gene prognostic model [40], and Libo Yang et al. established a nine-gene prognostic model [41]. In our study, we screened the genetic predictors using additional approaches, including univariate Cox, LASSO regression, and multivariate Cox analyses. Compared with the analysis performed by Yao Kang et al., the prognostic model presented here contains fewer prognostic-related genes and has higher prediction efficiency. Compared with the model established by Zihao Chen et al., the prognostic model presented here contains more prognostic genes but also more accurately predicts one- and three-year survival as measured by AUC values. Compared with the BLCA prognostic model established by Libo Yang et al., we performed in-depth filtering using the WGCNA method and used LASSO regression to optimize potential BLCA biomarkers. Compared with described BLCA prognostic models, our prognostic model has the advantages of rich data processing methods, fewer prognostic model-related genes, higher prediction efficiency, and higher model authenticity [42, 43]. Moreover, the accuracy of this prognostic model is as good as that of the classical bladder cancer prognostic model including FGFR3 and TP53 genes [44].

The relationships we revealed between the prognostic model and immune cells were confirmed in previous research studies. In this study, macrophage has a significant positive correlation with risk scores, which means it promotes bladder cancer metastasis and diffusion. While B cell's function is the opposite. Chen et al. found that LNMAT1 regulates bladder cancer lymphatic metastasis through CCL2-dependent macrophage recruitment [45]. Martinez et al. disclosed that BMP4 is closely associated with type II macrophage differentiation, promoting bladder cancer progression [46]. On the other hand, Zirakzadeh found that B cells are a vector for CD86 induction and inhibit the progression of bladder cancer [47]. These researches are in correspondence with our study.

In the labeled heatmaps, each row represents a module characteristic gene encoded by color, and the two columns represent clinical characteristics of the tumor and normal tissue, respectively. Each cell represents the Pearson correlation coefficient and *P* value (in parentheses) of the corresponding module characteristics, and the color of each cell represents the value of correlation.

The genes used in our BLCA prognostic model are involved in cancer. Gong Peng et al. regulated the intracellular *PDGFD* expression by controlling oxygen tension and found that *PDFGD* can stimulate glioblastoma proliferation [48]. *APOL1* is a risk factor for kidney and cardiovascular diseases [49, 50]. Recently, Jiewei Lin et al. found that *APOL1* activates the NOTCH1 signaling pathway to activate the proliferation and migration of pancreatic cancer cells [51]. In a Bolivian cohort study, high urine levels of *LYPD3* were found to be a cancer risk factor [52]. Guan-Rong Lai et al. found that *RPS6KA1* increases the sensitivity of prostate PC-3 cells to vitamin *D* and promotes the progression of prostate cancer [53]. *GNG7*, *MXRA7*, *ASB2*, and *CHMP4C* are also involved in the development of lung cancer, gastric cancer, colorectal cancer, and cervical cancer, respectively [54–57].

There are areas of this study that require additional investigation. While the theory of this study is sufficient and the prognostic model is worthy of subsequent clinical trials, whether the expression of the genes in the prognostic model change with the clinical progression of BLCA remains to be verified.

In conclusion, this study systematically constructed an eight-gene prognostic model of BLCA and revealed a moderate predictive effect on the prognosis and progression of BLCA. The specific mechanism of action and clinical application of the eight genes at the urinary bladder tissue level awaits further experimental exploration and clinical cohort verification.

## Figures and Tables

**Table 1:** Primers for quantitative real-time PCR (qRT-PCR).

Name	Forward	Reverse
GNG7	TTGAGCGCATCAAGGTCTCC	AAGGTTTCTTGTCCTTAAAGGGG
MXRA7	GAAGCTGAGGGGAAACCAGTAC	TCGGACATCTCGCCAAACGTCT
GAPDH	GACCCCTTCATTGACCTCAAC	CTTCTCCATGGTGGTGAAGA

## Data Availability

The datasets supporting the conclusions of this article are available in the TCGA (https://portal.gdc.cancer.gov) and GEO (https://www.ncbi.nlm.nih.gov/geo/). The datasets supporting the conclusions of this article are included within the article.

## Supplementary Materials

Supplementary Materials Figure S1: the flow chart of this study. Figure S2: results of gene coexpression network analysis in GSE188715. A: the soft threshold of the best scale-free topological model fitting index (left) and mean connectivity (right) were determined. The red horizontal line represents *R*2 = 0.9. B: gene clustering based on topological overlap matrix. Genes with relative correlation are located on the same or adjacent branches. Figure S3: TCGA and GTEx gene coexpression network analysis results. A: the soft threshold of the best scale-free topological model fitting index (left) and mean connectivity (right) were determined. The red horizontal line represents *R*2 = 0.9. B: gene clustering is based on a topological overlap matrix, and genes with relative correlation are located on the same or adjacent branches. Figure S4: ninety-five differential genes were screened by univariate Cox analysis of common significant genes in GSE188715, TCGA, and GTEx. All 95 genes are shown. The first four columns of the forest map are as follows: gene name, *p* value, hazard ratio, and 95% confidence interval, respectively. Table S1: clinicopathological features of bladder cancer patients.
