# Supplementary material for: Establishment and Validation of a Tumor Microenvironment Prognostic Model for Predicting Bladder Cancer Survival Status Based on Integrated Bioinformatics Analyses
Source: Evid Based Complement Alternat Med. 2022 Oct 3;2022:4351005. doi: 10.1155/2022/4351005 (PMC9550453; doi:10.1155/2022/4351005)
Supplement: Supplementary Materials — Figure S1: the flow chart of this study. Figure S2: results of gene coexpression network analysis in GSE188715. A: the soft threshold of the best scale-free topological model fitting index (left) and mean connectivity (right) were determined. The red horizontal line represents R2 = 0.9. B: gene clustering based on topological overlap matrix. Genes with relative correlation are located on the same or adjacent branches. Figure S3: TCGA and GTEx gene coexpression network analysis results. A: the soft threshold of the best scale-free topological model fitting index (left) and mean connectivity (right) were determined. The red horizontal line represents R2 = 0.9. B: gene clustering is based on a topological overlap matrix, and genes with relative correlation are located on the same or adjacent branches. Figure S4: ninety-five differential genes were screened by univariate Cox analysis of common significant genes in GSE188715, TCGA, and GTEx. All 95 genes are shown. The first four columns of the forest map are as follows: gene name, p value, hazard ratio, and 95% confidence interval, respectively. Table S1: clinicopathological features of bladder cancer patients. [file 4351005.f1.zip › Supplementary Table 1.docx]

**Supplementary Table 1. Clinicopathological features of bladder cancer patients**

| **Case number** | **Gender** | **Age** | **Stage** | **Grade** |
| --- | --- | --- | --- | --- |
| 1 | M | 58 | T1aN0M0 | L |
| 2 | M | 59 | T4aN0M0 | H |
| 3 | M | 70 | T2bN0M0 | H |
| 4 | M | 81 | T1N0M0 | L |
| 5 | F | 77 | T3aN0M0 | H |
| 6 | M | 80 | T2bN0M0 | L |
| 7 | M | 72 | T3aN0M0 | H |
| 8 | M | 70 | T2bN0M0 | H |
| 9 | M | 69 | T3aN0M0 | H |
| 10 | M | 72 | T2bN0M0 | H |
| 11 | F | 84 | T1N0M0 | L |
| 12 | M | 71 | T1N0M0 | H |
| 13 | F | 75 | T3aN0M0 | L |
| 14 | M | 75 | T2bN0M0 | H |
| 15 | M | 61 | T3aN0M0 | H |
| 16 | M | 74 | T2bN0M0 | H |
| 17 | F | 49 | T2aN0M0 | L |
| 18 | F | 78 | T2aN0M0 | L |
| 19 | M | 64 | T2aN0M0 | L |
| 20 | M | 82 | T2bN0M0 | H |
| 21 | F | 68 | T2aN0M0 | L |
| 22 | M | 70 | T2bN0M0 | H |
| 23 | F | 68 | T3aN0M0 | H |
| 24 | M | 66 | T1N0M0 | L |
| 25 | M | 65 | T4aN3M0 | H |

Stage: American Joint Committee on Cancer (AJCC) TNM system; Grade: 1998 World Health Organization and International Society of Urological Pathology grading system.
